# Delayed Macronutrients’ Target Achievement in Parenteral Nutrition Reduces the Risk of Hyperglycemia in Preterm Newborn: A Randomized Controlled Trial

**DOI:** 10.3390/nu15051279

**Published:** 2023-03-04

**Authors:** Maria Di Chiara, Gianluigi Laccetta, Daniela Regoli, Lucia Dito, Caterina Spiriti, Benedetta De Santis, Elisa Travaglia, Rita Prota, Pasquale Parisi, Roberto Brunelli, Giovanni Boscarino, Gianluca Terrin

**Affiliations:** 1 Department of Maternal and Child Health, Policlinico Umberto I, Sapienza University, 00161 Rome, Italy; 2 Department of Neuroscience, Mental Health and Sense Organs (NESMOS), Faculty of Medicine and Psychology, Sant’Andrea University Hospital, Sapienza University of Rome, 00189 Rome, Italy

**Keywords:** amino acids, energy, intake, growth, neonates, VLBW, EUGR

## Abstract

Hyperglycemia (HG) is an independent risk factor of mortality and morbidity in very low birth weight newborns (VLBW). Achievement of high nutritional intakes in the first days of life (DoL) by parenteral nutrition (PN) increases the risk of HG. We aim to assess if a delayed achievement of the PN macronutrient target dose could reduce the occurrence of HG in VLBW. We enrolled 353 VLBW neonates in a randomized controlled clinical trial comparing two PN protocols that differed in the timing of energy and amino acid target dose achievement: (1) early target dose achievement (energy within 4–5 DoL; amino acids within 3–4 DoL) vs. (2) late target dose achievement (energy within 10–12 DoL; amino acids within 5–7 DoL). The primary outcome was the occurrence of HG during the first week of life. An additional endpoint was long-term body growth. We observed a significant difference in the rate of HG between the two groups (30.7% vs. 12.2%, *p* = 0.003). Significant differences were observed in terms of body growth at 12 months of life between the two groups (weight Z-Score: −0.86 vs. 0.22, *p* = 0.025; length: −1.29 vs. 0.55, *p* < 0.001). Delayed achievement of energy and amino acid intake may be useful to reduce the risk of HG along with an increase of growth parameters in VLBW neonates.

## 1. Introduction

Despite improvement in neonatal care, post-natal growth restriction (EUGR) associated with undernourishment remain a challenge for neonatologists. Thus, adequate nutritional support immediately after birth is essential for preterm newborns to limit EUGR. Over the last decade, an early enhanced nutrition strategy has been adopted routinely in NICU in order to ensure a neonatal growth as close as possible to that of a fetus of the same gestational age (GA). In particular, current guidelines recommend preterm newborns to receive high doses of amino acids (i.e., 1.5–3.5 g/kg/d) and energy (i.e., 90–120 kcal/kg/d) [1,2].

However, the efficacy of this nutritional approach in terms of growth herein remains controversial [3,4,5]. Preterm neonates are considered to be susceptible to macronutrient deficits because of their limited glycogen and fat stores and increased energy and protein requirements to sustain growth. However, preterm neonates in the first week of life are in critical ill condition; therefore, they are probably unable to manage high macronutrient supplementation. The efficacy and safety of an enhanced PN in those critically ill newborns given early in life is largely undefined. In exclusively preterm individuals, current advice is to start parenteral intake as soon as possible after birth to avoid the metabolic derangement caused by the interruption of the continuous feeding in utero.

However, guidelines recommend administration of PN ensuring that it is advisable continuing to treat a child with PN being wary that it might become harmful for the child [6]. A growing body of literature has shown that enhanced PN is associated with side effects potentially harmful for preterm newborns. Stensvold et al. in an observational cohort study including 343 extremely low birth weight infants, demonstrated that enhanced PN during the first week of life was associated with hyperglycemia that was in turn associated with an independent risk factor of mortality [7]. A recent cohort study demonstrated that up to 98% of very low birth weight newborns (VLBW) developed at least one metabolic side effect associated with enhanced PN according to the current recommendations [8]. Randomized studies in adults and children demonstrated that the early use of PN in critically ill subjects negatively affected clinical outcomes [9,10] indicating that the harms of early PN support outweigh benefits in that population. In particular, in the preplanned sub analysis of the PEPaNIC trial including 209 critically ill term neonates, which were defined as undernourished on admission to PICU, showed that the use of early PN resulted in an increased rate of morbidity and reduced the length of PICU stay compared with withholding PN for 1 week (late PN) [11].

We hypothesized that delaying the time needed to achieve the macronutrient target of supplemental PN beyond the critical window period (first week of life), even maintaining the same target value recommended, may reduce the occurrence of metabolic side effects in very low birth weight infants (VLBW). The effects of this kind of nutritional strategy on growth in VLBW infants still needs to be established. 

To test this hypothesis, we designed a controlled clinical trial aiming to investigate the efficacy and safety of two PN strategies, which were different for the time of target achievement: (1) Group 1: early target achievement of PN; (2) Group 2: delayed target achievement of PN. 

## 2. Materials and Methods

### 2.1. Standard Protocol Approval, Ethics and Patient Consent

The study is a single center prospective randomized controlled trial. 

The study was conducted in conformity with World Medical Association Declaration of Helsinki for medical research involving human subjects; it was approved by Ethics Committee of Policlinico Umberto I, University La Sapienza of Rome (number 5089). We obtained written informed consent from all parents. 

### 2.2. Population and Randomization

We included all newborns with a gestational age (GA) <32 weeks or body birth weight (BW) <1500 g, consecutively admitted to the NICU of Policlinico Umberto I, La Sapienza University of Rome over a 4-year period. We excluded from the analysis neonates with major congenital malformations, inborn errors of metabolism, congenital infections, loss of central lines, hospital discharge or transfer or death within 24 h of life.

Consecutive eligible patients were randomly assigned (1:1) to either one of the two PN protocols characterized by early (Group 1) or delayed (Group 2) achievement of the macronutrient target intake.

Target intake was defined by the sum of macronutrient intake received by parenteral plus enteral nutrition. In both groups, the enteral nutrition protocol was administered according to a unique protocol. 

The nutritional protocol followed by patients included in the two study groups were reported in Table 1. 

Patients were allocated to each group according to a software generated randomization list. 

Concealment of group assignment was ensured by the use of a computerized randomization system. On the randomization list, each patient was associated with a code belonging to either one of the two treatment protocols. A neonatologist in charge of PN prescription had to insert the specific code in the software that opened up either one of the group treatments. 

Performing a double-blind study would not have been ethically feasible, physicians prescribing the supplemental PN proposed by the software were unaware of the study aims and we used the third-party blind observer method to assess efficacy. Outcome assessors and investigators who were not directly involved in NICU patient care were not informed of treatment allocation. 

### 2.3. Outcome

The primary outcome measure was the rate reduction of patients experiencing severe hyperglycemia (HG) between Group 1 (HG 30%) and Group 2 (15%) during the first week of life. 

### 2.4. Nutritional Protocol

In both groups, PN was initiated within 24 h after admission into the NICU. 

The macronutrient dose varied according to group assignment (Table 1). 

Neonatologists in charge prescribed PN on a daily basis to preterm neonates based upon each clinical condition, laboratory results and weight by using a specific software. The requirement of macro- and micronutrients administered through PN was calculated by deducting the amount of enteral nutrition from the total energy requirement. 

The PN was administered via central vascular access. The overall fluid intake administered by combined EN and PN started with 70–90 mL/kg/day and increased by 10–20 mL/kg/day until the achievement of 150–180 mL/kg/day. The enteral feeding scheme was the same for the 2 study groups. Minimal enteral feeding was commenced within 48 h after birth at 10–20 mL/kg/day. The amount was increased by 20–30 mL/kg/day if enteral nutrition (EN) was tolerated. We withheld enteral feeding in case of severe abdominal distension, emesis, ileus with visible intestinal loops, blood in the stools, apnea, bradycardia, inadequate perfusion and hemodynamic instability. Human milk (HM) of own mother without fortifications was given fresh as soon as possible after birth if available. The preterm formula (PF) was administered to the infants when HM was not available or sufficient. Preterm HM was assumed to contain 65 Kcal/100 mL (1.5 g of protein/100 mL, 3.5 g of fat/100 mL and 6.9 g of carbohydrate/100 mL). Macronutrient contents of formula (Pre-Nidina Nestlè 1, Milan, Italy) and of PN were calculated based on the published manufacturer’s labels and included proteins (TrophAmine1 6% Braun Medical Inc., Irvine, CA, USA), lipids (Smoflipd 1, Fresenius Kabi, Lake Zurich, IL, USA) and carbohydrates (Dextrose injection 10%, Fresenius Kabi, USA) expressed in g/kg/day.

### 2.5. Data Collection

All patient data were stored in a logged database that was closed 90 days after enrollment of the last patient. 

The HG was defined as two consecutive blood glucose levels greater than 180 mg/dL at least 3 h apart. Blood glucose levels were monitored by a validated micro-method from capillary blood and analyzed by point of care device Accu-Chek Inform II glucometer (Roche, Indianapolis, IN, USA) four to eight times per day from the first days of life and less frequently when the clinical conditions were stabilized [12]. In case of HG occurrence, the treatment consisted of reducing the intravenous glucose concentration by 1–2 mg/kg/min. In case of failure of blood glucose reduction within 4 h, the dextrose was again decreased until a minimum of 5 mg/kg/min. To do this, we reduced velocity volume of the prescribed PN, and thus also amino acids and lipids were decreased. 

Investigators collecting reporting forms were blinded to the assigned group.

Medical staff were blinded to the study aims but not to eligibility criteria. Researchers not involved in the clinical practice provided information to the parents and collected all data useful for statistical analysis. A third-party observer, not involved in the previous steps, was involved to collected data on the primary outcome. A blinded statistician performed data analysis. 

We prospectively recorded prenatal, perinatal, and postnatal data in a specific data form. Data on PN, EN and feeding tolerance were collected daily. Data on daily cumulative parenteral and enteral nutritional intake were reported in a specific data form. 

In order to study the occurrence of PN related complications, we collected data on glycemia, hypercalcemia, hypo-phosphoremia and hyponatremia. 

We also collected data on survival and morbidities of prematurity. Overall morbidity was defined as the presence of at least one of the major prematurity complications: necrotizing enterocolitis (NEC) Bell’s stage ≥ 2, intraventricular hemorrhage (IVH) stage ≥ 2, periventricular leukomalacia (PVL), culture proven sepsis, retinopathy of prematurity (ROP) stage ≥ 3 and bronchopulmonary dysplasia (BPD). Diagnosis of NEC, BPD, IVH, PVL, ROP and culture proven sepsis were performed according to standard criteria by physicians unaware of the study design and aims, as previously described [13,14]. Growth parameters were also collected during the study period. Nurses unaware of the study aims measured growth parameters. Standardized percentiles and standardized Z-score measures of body weight, length and CC were calculated adopting Ines chart and intergrowth charts [15]. Body weight, length and CC were recorded daily from birth to 36 weeks of postmenstrual age (PMA) at 12 and 24 months [16,17]. We defined EUGR as the reduction > 1SD (−1.28) in anthropometric parameters Z-Score between birth and 36 weeks of PMA [18]. 

### 2.6. Statistical Analysis

Statistical analysis performed using Statistical Package for Social Science Software for Microsoft Windows (SPSS Inc., Chicago, IL, USA), version 27.0. We checked for normality using a Shapiro–Wilk test. The mean and standard deviation or median summarized continuous variables. We used a chi-square test for categorical variable, *t*-test, Mann–Whitney and Wilcoxon test for paired and unpaired variables. The analysis of the primary outcome was performed per intention to treat. Patients lost to follow up before the primary endpoint were considered as having HG. We performed a binary regression analysis to study the possible influence of confounding variables on the occurrence of HG. The influence of covariates on the occurrence of mortality and morbidity rate was evaluated by logistic regression analysis. After checking for assumptions, a linear regression analysis with a stepwise method was performed to investigate the influence of covariates on growth at 12 months. The level of significance for all statistical tests was 2 sided (*p* < 0.05). The statistician was blinded to study aims and the patient codes were revealed after statistical analysis. 

We estimated the need of 177 participants in each group to obtain a power of 90% (type 1 error = 0.05, two tailed test, drop out 10%). 

## 3. Results

**Figure 1:**
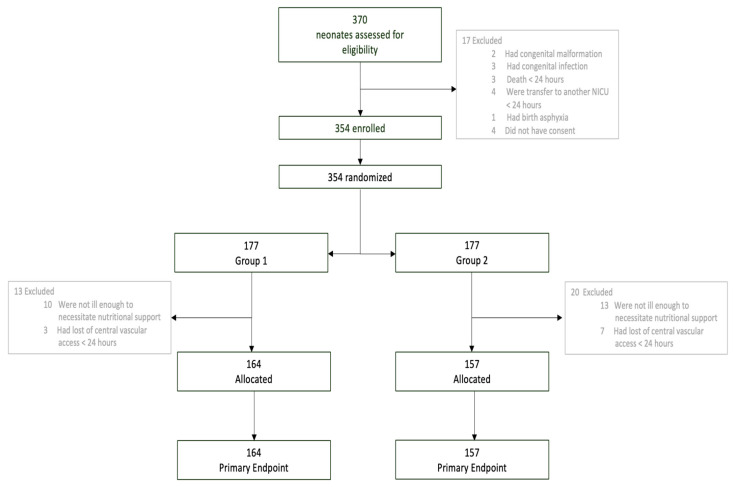
Screening and randomization.

A total of 321 patients underwent randomization and were included in the analysis, (Figure 1). 

At baseline, the characteristics of the patients were similar between the two groups, (Table 2). 

**Figure 2:**
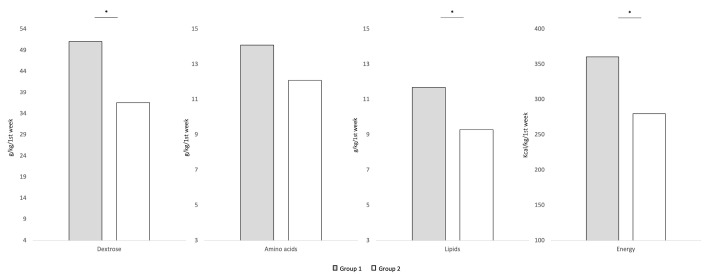
Macronutrient intake administered through PN during the first week of life. Figure legend: * *p*-value < 0.05.

Group 1 showed a significantly decreased duration of PN compared with Group 2 (13.43 DoL vs. 17.65 DoL; *p* = 0.018). Time to reach full enteral feeding (FEF) was similar between the two study groups (26.1% vs. 30.2%; *p* = 0.249). Duration of vascular access was significantly (*p* < 0.001) lower (9.9, 8.0 a 11.8 DoL) in Group 1 compared with Group 2 (19.3, 15.8 to 23.5 DoL). Figure 2 reports macronutrient intakes of neonates included in the study. Group 1 showed a significant increased dextrose, lipids and energy intake received through PN within the first week of life compared with Group 2 (Figure 2). 

**Figure 3:**
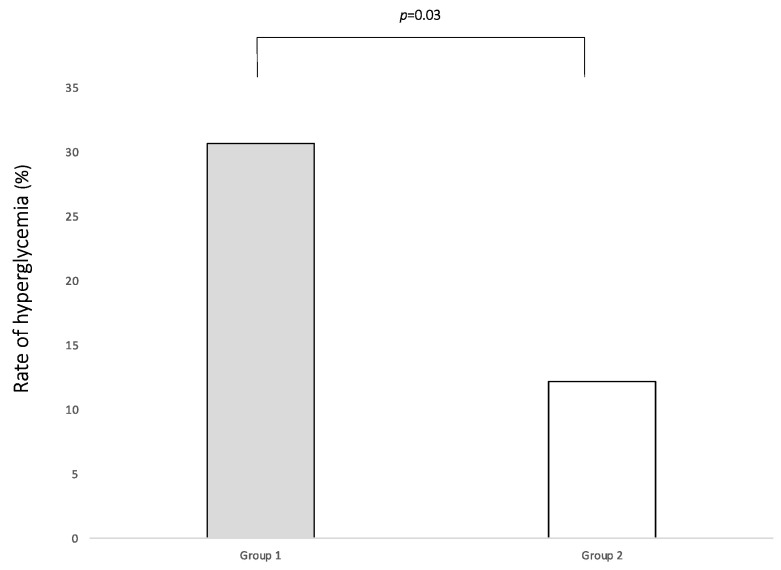
Rate of occurrence of hyperglycemia in the two study groups during the first week of life.

The rate of HG within 0–7 DoL is reported in Figure 3. 

The rate of hypoglycemia within 0–7 DoL was similar between the two groups (12.4 vs. 12; *p* = 0.524). The rate of increase of the separate macronutrients (dextrose, amino acids and lipids) are reported in Supplementary Materials (Figures S1–S3). 

Data analyzed also per protocol showed similar results on the primary outcome. Despite the overall morbidity not being different among groups, we observed that the rate of BPD and ROP were significantly higher in Group 1 than Group 2 (Table 3). Whereas the rate of culture-proven sepsis was significantly lower in Group 1 compared to Group 2 (Table 3).

The binary logistic regression analysis showed that group assignment did not influence the risk of BPD (Table S1). The binary logistic regression analysis showed that group assignment independently influenced the risk of ROP, along with GA and blood transfusion (Table S2). The binary logistic regression analysis showed that group assignment independently influenced the risk of culture-proven sepsis along with CVA (Table S3).

In Table 4 and Table 5, we reported the results of the multivariate analysis. 

We found that group assignment (ß = 1.095, *p* = 0.000) along with GA (ß = 1.765, *p* = 0.004) and culture-proven sepsis (ß = 1.503, *p* = 0.000) significantly influenced the risk of HG (Table 4). The binary logistic analysis showed that HG independently influenced the risk of mortality (Table 5). 

The rate of EUGR was lower in Group 1 compared with Group 2 (63.4 vs. 75.5, *p* = 0.035). In Table 6, we reported results regarding growth at 12 months of life for the enrolled infants. 

Unstandardized BW, length and CC were reduced in Group 1 compared with Group 2 at 12 months of life (Table 6). We observed significant difference in the Z-Score of weight and length between the two Groups (Table 6); Group 1 showed a significantly decreased Z-score for both weight and length parameters compared to Group 2 (Table 6). 

Linear regression analysis showed that only actual energy intake in the first week of life through PN were positively related with weight at 12 months of life, (Table 7).

## 4. Discussion

Delayed target achievement of recommended nutritional intake reduces the risk of HG in preterm newborns. The occurrence of HG in the first week of life is an independent risk factor for mortality during hospitalization. Additionally, early target achievement reduces the risk of sepsis but is associated with an increased risk of ROP. Contrasting effects on brief- and long-term growth were observed comparing the two nutritional strategies adopted in the trial. If PN strategy characterized by early introduction of macronutrients was associated with an improvement in short-term growth, the analysis of long-term growth parameters revealed that delayed target achievement was advantageous for body weight and length as showed in multivariate model analysis.

The drawbacks of an early target achievement seem to overcome the related advantages. We observed that the amino acid intake within the first week of life was similar between the two groups, whereas the amount of dextrose and lipids during the first week of life were significantly different between Group 1 and Group 2. Thus, it is plausible that the related effects of a nutritional strategy characterized by delayed target achievement may depend on the amount of nonprotein calories. 

Previous studies comparing PN strategies focused mainly on the effects of maximum macronutrient target intake but not on the modalities of advancement of macronutrient intake by PN. So far, in critically ill newborns, there is a dearth of adequately powered, randomized, controlled trials that address the effects of PN on clinical outcomes. Previous recommendations for the optimal amount and composition of PN were based on very few observational studies on infants in critical conditions, in which PN support was advised without focusing on the facts that critically ill infants face different clinical phases during NICU hospitalization [2,19]. Macronutrient metabolism in neonates may be different in the critical phase compared to the stable phase. Therefore, the nutritional approach should be modulated on the basis of the clinical condition of newborns [2,20]. 

The PEPaNIC trial showed that starting PN after 1 week in critically ill children is clinically superior to providing early PN as soon as possible after hospitalization [9]. Recently, Van Puffelen et al. in a sub analysis of the PEPaNIC trial demonstrated the efficacy and safety of withholding supplemental parenteral nutrition for 1 week in all neonates enrolled in the trial [21]. However, that RCT included only newborn babies aged up to 28 days and Authors compared an early versus delayed PN characterized by the same amount of macronutrients. 

Our results are in keeping with those of Stensvold et al. whereby, in a prospective cohort study comparing enhanced PN with standard PN, HG is an independent risk factor of mortality in preterm newborns [7,22]. 

Our finding of greater morbidity in neonates receiving the early PN target is in keeping with previous evidence in critically ill adults, children and newborns, which has led to calls for the deimplementation of early PN in these groups [10]. In accordance with our results, Uthaya et al., in an observational study evaluating an early versus later introduction of PN in very preterm infants, found evidence of higher rates of morbidity, including ROP, in the group who received early PN [23]. In good agreement, we reported an increased risk of ROP in the early target group. Many studies have demonstrated that hyperglycemia may be correlated with an increased risk of morbidity of ROP in premature infants [24,25]. The latest meta-analysis integrated all relevant research and investigated the association between HG and the development of ROP in premature infants [26]. This meta-analysis demonstrates that preterm infants with hyperglycemia have a tendency to have an increased risk of ROP. The mechanism of how hyperglycemia affects ROP is still inconclusive. Another possible pathogenetic factor of ROP could be identified in blood transfusions [27]. It has been thoroughly described that fresh-frozen plasma from adult donors administered to VLBW represents an actual source of IGF-1, which plays a main role in the pathogenesis of ROP [28]. Our multivariate analysis confirmed this hypothesis. 

Despite our data disconfirm the relation between early PN intake and BPD occurrence, this aspect is still an ongoing debate. Several retrospective studies reported the association between low caloric intake and BPD development. Malnutrition seems to worsen BPD probably by compromising lung development and function; a recent study found that preterm infants who developed BPD received low caloric intake [29]. Our findings do not argue the recommended energy target but the time to achieve target doses through PN. We found that a delayed target achievement of parenteral macronutrients might be a protective factor for BPD in preterm neonates. In any case, it is possible to speculate that this finding could be related to the effects of HG. It has been demonstrated that HG induces an increased proinflammatory cytokine response in the blood of preterm and term neonates [30]. High glucose levels lead to increased oxidative stress and activate caspase with consequent reactive oxygen species (ROS) production, which are in turn known to be involved in the pathogenesis of BPD. 

The finding of increased sepsis in the delayed target group is out of keeping with the outcomes in children comparing early versus late PN [9,11]. The main reason for this may be that the slow target achievement resulted in a longer duration of PN leading to the longer keeping of CVA. PN carries the well described risk of blood infection [31]. Thus, the increase in sepsis may be due to the longer permanence of CVA. 

An early target achievement PN resulted in a reduction of EUGR occurrence. Previous studies have shown the association between early PN and improved short-term growth in preterm newborns [3]. The RCTs demonstrated a better brief-term growth in newborns receiving a high-energy intake [32,33]. Comparative trials have not clarified the role of energy intake, in particular the optimal protein–energy ratio, and the adjusting dose of that ratio along with the progression of PN in VLBW is yet to be determined [6,34]. Our study focused on the effects of early energy intake on long-term growth in VLBW neonates. The vast majority of available evidence assessing long-term growth parameters compared solely the amino acids intake solution but not the energy intake [3,5]. Thus, our study provides additional insight into long-term growth measures resulting from differences in the only energy intake in VLBW neonates.

Despite interesting results, our study had some limitations. Parents of neonates included in the study and neonatologists were aware of the group assignment. Individualized PN solution adjusting is the milestone of our policy on PN in order to avoid deleterious consequences of PN-related complications. Despite being a potential information bias, we have preferred that neonatologists in charge knew the composition of PN in order to easily adjust the PN prescription in case of metabolic complications related to PN administration. To limit selection bias, physicians evaluating eligibility were blinded to the study aims and used objective inclusion criteria (such as GA and BW). To limit observer bias, outcome assessors were unaware of group assignment. We discussed and defined a protocol for the collection, measurement and interpretation of data before starting the study. Finally, a blinded statistician performed the data analysis.

The overall morbidity and mortality were higher in Group 2, and it is very close to statistical significance. Further, Group 2 had a significantly higher risk for sepsis. However, we performed a multivariate analysis that identified hyperglycemia as independent risk factor for mortality in a model including sepsis. Additionally, we speculate that overcoming EUGR at 12 months of life could be explained by the increased morbidities, which in turn limit neonates’ growth. To verify this hypothesis, we performed a linear regression analysis showing that energy intake independently influences growth at 12 months of life.

The trial was not designed for evaluating the difference in long-term growth; thus, the sample power of the study is insufficient to evaluate differences between study groups in body weight at 12 months of life.

## 5. Conclusions

HG represents one of the most common complications of early PN, in preterm newborns. The adoption of a nutritional protocol characterized by a delayed target macronutrient achievement may contribute to reducing the risk of HG. The benefit observed on short-term growth might not outweigh the harm of having increased complications related of early enhanced PN. Thus, it should be advisable to achieve macronutrient targets with more caution.

## Figures and Tables

**Table 1:** Nutritional protocol of neonates included in the two study groups.

	Birth Weight < 1000 g		Birth Weight ≥ 1000 g	
	Group 1	Group 2	Group 1	Group 2
Energy (kcal/kg/day)				
Starting dose (min–max)	45–58	45–58	40–45	40–45
Target dose (min–max)	100–110	100–110	90–100	90–100
Time of target achievement, days	4–5	10–12	4–5	10–12
Amino acids (g/kg/day)				
Starting dose (min–max)	2.0–2.5	2.0–2.5	3.2–3.5	3.2–3.5
Target dose (min–max)	3.5–4.0	3.5–4.0	3.5–4.0	3.5–4.0
Time of target achievement, days	3–4	5–7	3–4	5–7
Dextrose (g/kg/day)				
Starting dose (min–max)	6.5–7.0	6.5–7.0	6.5–7	6.5–7.0
Target dose (min–max)	13.0–14.0	13.0–14.0	13.0–14.0	13.0–14.0
Time of target achievement, days	5–7	10–12	5–7	10–12
Lipids (g/kg/day)				
Starting dose (min–max)	1.5–2.0	1.5–2.0	1.0–1.5	1.0–1.5
Target dose (min–max)	3.5–4.0	3.5–4.0	3.0–3.5	3.0–3.5
Time of target achievement, days	3–5	5–7	3–5	5–7

**Table 2:** Baseline clinical characteristics of neonates enrolled in the study.

	Group 1 *n* = 164	Group 2 *n* = 157	*p*
*Pre-natal characteristics*			
Antenatal corticosteroids ^1^, No. (%)	112 (69.6)	105 (70.0)	0.516
IUGR ^2^, No. (%)	24 (14.9)	28 (18.9)	0.215
Pregnancy-induced hypertension, No. (%)	38 (23.6)	30 (19.9)	0.254
Hypothyroidism, No. (%)	20 (12.4)	21 (14.1)	0.395
Twins, No. (%)	51 (31.1)	53 (34.6)	0.291
Gestational Diabetes, No. (%)	16 (9.9)	21 (14.5)	0.149
Mother’s age ≥ 35 years old, No. (%)	65 (43.9)	69 (45.4)	0.444
Cesarean section, No. (%)	142 (86.6)	132 (86.3)	0.533
*Perinatal characteristics*			
Gestational age, weeks	29.4 (29.0 to 29.8)	29.6 (29.2 to 30.0)	0.572
Birth weight, g	1225.7 (1170.4 to 1280.9)	1301.9 (1241.4 to 1362.3)	0.067
Male sex, No. (%)	88 (53.7)	80 (51.6)	0.400
SGA ^3^, No. (%)	37 (23)	25 (16.9)	0.116
ELBW ^4^, No. (%)	43 (26.2)	30 (19.1)	0.083
5-min Apgar score	7.8 (7.6 to 8.0)	7.9 (7.6 to 8.1)	0.483
pH at birth	7.3 (7.2 to 7.3)	7.3 (7.2 to 7.3)	0.851
Base excess on cord blood, mmol/L	−5.2 (−5.7 to −4.6)	−5.9 (−6.8 to −5.2)	0.107
CRIB II score ^5^	6.4 (5.8 to 7.0)	5.7 (4.9 a 6.5)	0.176
Length of hospital stay, days	63.8 (57.8 to 69.8)	58.3 (53.4 to 63.3)	0.167

**Notes**: ^1^ An intramuscular steroid cycle in two doses of 12 mg over a 24 h period; ^2^ IUGR: intrauterine growth restriction; ^3^ SGA: small for gestational age; ^4^ ELBW: extremely low birth weight; ^5^ CRIB II score: clinical risk index for babies. Data were expressed as mean (lower to upper limits 95% confidence interval) when not specified.

**Table 3:** Morbidities of newborns included in the study.

	Group 1	Group 2	*p*
	*n* = 164	*n* = 157	
**Mortality,** No. (%)	9 (5.5)	9 (5.7)	0.558
**NEC stage III,** No. (%)	1 (0.6)	4 (3.0)	0.125
**BPD,** No. (%)	13 (7.9)	4 (2.7)	0.033 *
**Culture-proven sepsis,** No. (%)	12 (7.4)	28 (18.7)	0.002 *
**Retinopathy of prematurity,** No. (%)	34 (20.9)	16 (10.5)	0.009 *
**Periventricular leukomalacia,** No. (%)	4 (2.5)	4 (2.9)	0.552
**Intraventricular hemorrhage,** No. (%)	12 (7.4)	10 (6.7)	0.493
**Patent ductus arteriosus,** No. (%)	39 (23.9)	35 (23.2)	0.491
**Severe anemia,** No. (%)	43 (26.4)	41 (27.2)	0.489
**Overall morbidity,** No (%)	51 (31.1)	62 (40.3)	0.056

**Notes.** * *p* < 0.05.

**Table 4:** Binary logistic regression analysis to evaluate the influence of covariates on the occurrence of hyperglycemia.

Variables	ß	S.E.	Wald	*p* Value	Odds Ratio (OR)	95 C.I for OR	
						Lower	Upper
**GA** °	1.765	0.618	8.166	0.004 *	5.840	1.74	19.59
**Group assignment**	1.095	0.310	1.492	0.000 *	1.038	1.62	5.48
**Antenatal corticosteroids** ^±^	0.037	0.310	0.014	0.905	1.038	0.56	1.90
**Culture-proven sepsis**	1.503	0.393	14.492	0.000 *	4.496	2.08	9.71

**Notes.** ° GA = gestational age < 32 weeks; ^±^ Intramuscolar steroid cycle in wo doses of 12 mg over a 24-h period. * *p* < 0.05.

**Table 5:** Binary logistic regression analysis to evaluate the influence of covariates on the occurrence of mortality.

Variables	ß	S.E.	Wald	*p* Value	Odds Ratio (OR)	95 C.I for OR	
						Lower	Upper
**GA** °	1.867	1.077	3.003	0.083	6.467	0.783	53.418
**Group assignment**	0.266	0.285	0.876	0.349	1.305	0.747	2.280
**Hyperglycemia**	1.620	0.602	7.229	0.007 *	5.052	1.551	16.452
**Sepsis** ^±^	0.655	0.607	1.165	0.280	1.926	0.586	6.332
**BPD** ^ξ^	0.432	0.743	0.338	0.561	1.541	0.359	6.613

**Notes.** ° GA = gestational age < 30 weeks; ^±^ Culture-proven sepsis; ^ξ^ BPD: bronchopulmonary dysplasia. * *p* < 0.05.

**Table 6:** Growth at 12 months of life in the two groups of study.

Growth at 12 Months	Group 1	Group 2	*p*-Value
Unstandardized parameters			
Weight, g	8691 (8403 to 8978)	9309 (8944 to 9674)	0.010 *
Length, cm	72.76 (72.01 to 73.50)	74.58 (73.59 to 75.57)	0.004 *
CC, cm	45.19 (44.87 to 45.51)	45.86 (45.44 to 46.28)	0.013 *
BMI, Kg/m²	16.40 (15.97 to 16.83)	16.63 (16.20 to 17.05)	0.492
Standardized Percentiles			
Weight, g	37.55 (31.63 to 43.47)	56.00 (47.97 to 64.03)	0.000 *
Length, cm	39.79 (33.28 to 46.30)	64.68 (56.57 to 72.78)	0.000 *
CC, cm	46.44 (40.34 to 52.53)	67.44 (60.44 to 74.44)	0.000 *
Standardized Z-Score			
Weight, g	−0.86 (−1.58 to −1.15)	0.22 (−0.91 to 0.53)	0.025 *
Length, cm	−1.29 (−1.97 to −0.60)	0.55 (0.22 to 0.88)	0.000 *
CC, cm	1.57 (0.50 to 2.64)	0.66 (0.39 to 0.93)	0.196
BMI, Kg/m²	−0.37 (−0.79 to 0.03)	−0.07 (−0.37 to 0.22)	0.298
Weight/Length, kg/cm	−0.17 (−0.38 to 0.03)	0.01 (−0.28 to 0.30)	0.315

**Notes**. * *p* < 0.05.

**Table 7:** Linear regression analysis of covariates on growth at 12 months.

Weight *Z-Score*	T	S.E.	ß		*p* Value	95 C.I for OR
					Lower	Upper
Model 1						
**Gestational age**	−0.464	0.158	−0.405	0.004	−0.776	−0.151
**Birth weight *Z-Score***	−0.093	0.301	−0.035	0.759	−0.688	0.503
**Energy intake ^1^**	−0.008	0.002	−0.594	0.000 *	−0.012	−0.004
Model 2						
**Gestational age**	−0.450	0.137	−0.393	0.001 *	−0.720	−0.180
**Weight 36 wks *Z-Score***	−0.062	0.242	−0.270	0.797	−0.541	0.416
**Energy intake ^1^**	−0.008	0.002	−0.585	0.000 *	−0.011	−0.004

**Notes.** ^1^ Actual energy intake 0–7 DoL (Kcal/kg). * *p* < 0.05.

## Data Availability

The datasets analyzed during the current study are available from the corresponding author on reasonable request.

## Supplementary Materials

The following supporting information can be downloaded at: https://www.mdpi.com/article/10.3390/nu15051279/s1. Figure S1: Rate of increase of dextrose, within the first week of life, in the two study groups; Figure S2: Rate of increase of amino acids within the first week of life in the two study groups; Figure S3: Rate of increase of lipids within the first week of life in the two study groups. Table S1. Binary logistic regression analysis to evaluate the influence of covariates on the occurence of bronchopulmonary dysplasia. Table S2. Binary logistic regression analysis to evaluate the influence of covariates on the occurence of ROP. Table S3. Binary logistic regression analysis to evaluate the influence of covariates on the occurence of Sepsis.
