# Supplementary material for: Delayed Macronutrients’ Target Achievement in Parenteral Nutrition Reduces the Risk of Hyperglycemia in Preterm Newborn: A Randomized Controlled Trial
Source: Nutrients. 2023 Mar 4;15(5):1279. doi: 10.3390/nu15051279 (PMC10005207; doi:10.3390/nu15051279)
Supplement: Supplementary file 1 [file nutrients-15-01279-s001.zip › nutrients-2134720-supplementary.pdf]

Supplementary Materials

Table S1. Binary logistic regression analysis to evaluate the influence of covariates on the occurence of bronchopulmonary dysplasia.

Variables	ß	S.E.	Wald	*p* value	Odds ratio (OR)	95 C.I for OR	
						Lower	Upper
ELBW °	2.749	0.818	11.283	*0.001**	15.625	3.14	77.70
IUGR±	-4.05	0.905	0.200	*0.655*	0.667	0.11	3.93
Male sex	0.039	0.600	0.427	*0.513*	1.480	0.457	4.80
Group assignment	1.050	0.664	2.496	*0.114*	2.857	0.777	10.506
Culture-proven sepsis	0.048	0.734	0.004	*0.948*	1.049	0.25	4.42
Mechanical ventilation$^{\xi}$	2.138	0.840	6.479	*0.011*	8.48	1.63	44.00

Notes. ELBW = birth weight < 1000 grams; ±IUGR = intrauterine growth restriction; $^{\xi}$ duration > 7 DoL
*$p < 0.05$

Table S2. Binary logistic regression analysis to evaluate the influence of covariates on the occurence of retinopathy of prematurity.

Variables	ß	S.E.	Wald	*p* value	Odds ratio (OR)	95 C.I for OR	
						Lower	Upper
GA °	1.872	0.564	11.018	*0.001**	6.500	2.152	19.630
IUGR±	0.092	0.508	0.033	*0.856*	1.096	0.405	2.97
Group assignment	-0.923	0.389	5.623	*0.018**	0.397	0.185	0.852
Culture-proven sepsis	0.578	0.472	1.502	*0.220*	1.782	0.707	4.492
Blood transfusion$^{\xi}$	1.542	0.379	16.586	*0.000**	4.673	2.225	9.813

Notes. °GA = gestational age < 30 weeks; ± IUGR = intrauterine growth restriction; $^{\xi}$at least one during hospital stay. *$p < 0.05$

Table S3. Binary logistic regression analysis to evaluate the influence of covariates on the occurence of sepsis.

Variables	ß	S.E.	Wald	*p* value	Odds ratio (OR)	95 C.I for OR	
						Lower	Upper
ELBW °	0.733	0.446	2.699	*0.100*	2.082	0.868	4.995
IUGR±	0.103	0.496	0.043	*0.835*	1.109	0.419	2.934
Antenatal corticosteroids$^{\xi}$	-0.323	0.392	0.679	*0.410*	0.724	0.336	1.560
Group assignment	1.386	0.405	11.682	*0.001*	3.998	1.806	8.852
CVA$^{\delta}$	1.837	0.640	8.245	*0.004**	6.279	1.792	22.004

Notes. °ELBW = birth weight < 1000 grams; ±IUGR = intrauterine growth restriction; $^{\xi}$ An intramuscular steroids cycle in two doses of 12 mg over a 24-hour period; $^{\delta}$CVA = central vascular access > 7 DoL. *$p < 0.05$

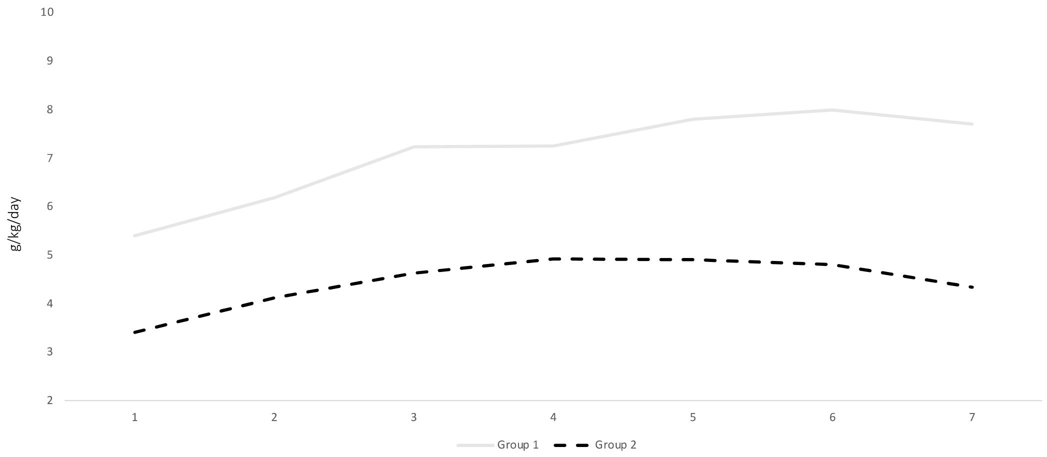

Figure S1: rate of increase of dextrose, within the first week of life, in the two study Groups.

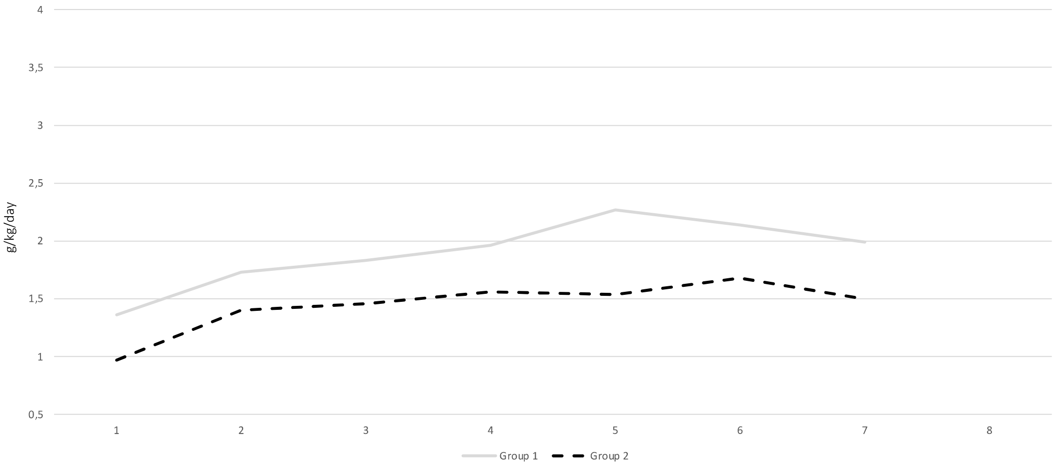

Figure S2: rate of increase of amino acids, within the first week of life, in the two study Groups.

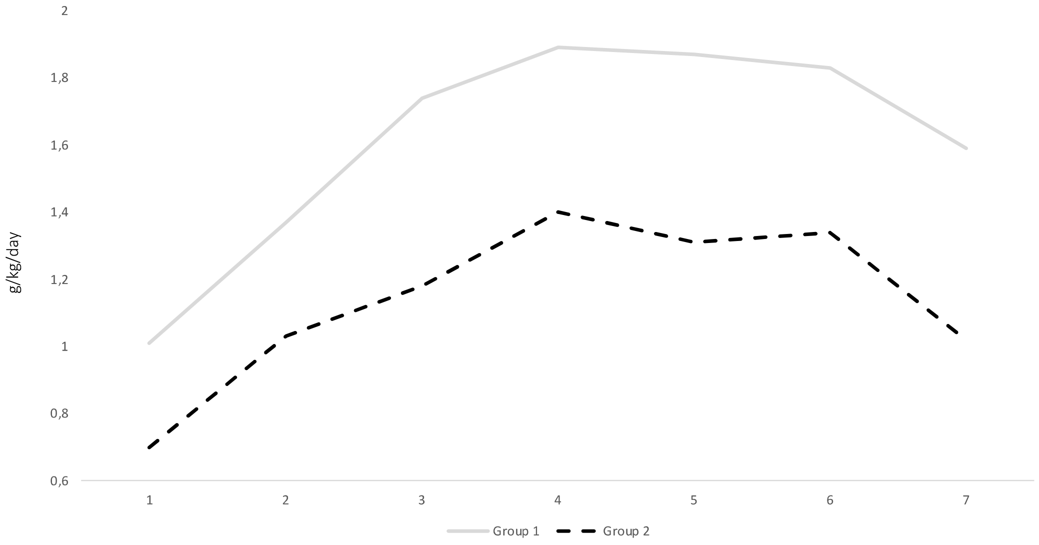

Figure S3: rate of increase of lipids, within the first week of life, in the two study Groups.
